# Cryo-EM as a tool to study bacterial efflux systems and the membrane proteome

**DOI:** 10.12703/r/10-24

**Published:** 2021-03-01

**Authors:** Philip A Klenotic, Christopher E Morgan, Edward W Yu

**Affiliations:** 1 Department of Pharmacology, Case Western Reserve University School of Medicine, 2109 Adelbert Rd, Cleveland, OH 44106-4965, USA

**Keywords:** Antibiotic resistance, RND-type transporters, cryo-electron microscopy

## Abstract

Antibiotic resistance is an emerging threat to global health. Current treatment regimens for these types of bacterial infections are becoming increasingly inadequate. Thus, new innovative technologies are needed to help identify and characterize novel drugs and drug targets which are critical in order to combat multidrug-resistant bacterial strains. Bacterial efflux systems have emerged as an attractive target for drug design, as blocking their export function significantly increases the potency of administered antibiotics. However, in order to develop potent and tolerable efflux pump inhibitors with high efficacy, detailed structural information is required for both the apo- and substrate-bound forms of these membrane proteins. The emergence of cryo-electron microscopy (cryo-EM) has greatly advanced the field of membrane protein structural biology. It has significantly enhanced the ability to solve large multi-protein complexes as well as extract meaningful data from a heterogeneous sample, such as identification of several assembly states of the bacterial ribosome, from a single data set. This technique can be expanded to solve the structures of substrate-bound efflux pumps and entire efflux systems from previously unusable membrane protein sample preparations. Subsequently, cryo-EM combined with other biophysical techniques has the potential to markedly advance the field of membrane protein structural biology. The ability to discern complete transport machineries, enzymatic signal transduction pathways, and other membrane-associated complexes will help us fully understand the complexities of the membrane proteome.

## Introduction

The widespread prevalence of multidrug-resistant (MDR) bacteria is an emerging threat to global health. Data released by the Centers for Disease Control and Prevention show that in the United States there are more than 2.8 million new cases of antibiotic-resistant infections each year^1^ with considerably more worldwide^2^. With the continual exposure of antibiotics given in standard treatment regimens, MDR, extensively drug-resistant (XDR), and totally drug-resistant (TDR) bacterial strains have emerged^3,4^. These drug-resistant strains are especially problematic in hospital settings, where immunocompromised and other high-risk patients are extremely susceptible to bacterial infections. This is highlighted by a recent analysis of lungs harvested for use in transplant procedures; almost 60% of donor lung tissue had bacterial contamination, and about 5% of those contained MDR strains^5^. Whereas these pathogens currently can be eliminated with a tailored antibiotic treatment, it is well established that hospital-borne MDR bacteria such as *Acinetobacter baumannii*^6–8^, *Staphylococcus aureus*^9–11^, and *Escherichia coli*^12–15^ can develop resistance mutations through natural evolution, leading to the continual need to develop new antibiotic treatments. To help combat these superbugs, unique antibiotics and potent inhibitors of critical bacterial survival systems need to be developed. A common method for identification of new biocides is to conduct a blind screen for potential compounds from established chemical libraries and then test them in *in situ* bacterial systems^16,17^. The binding capabilities of these compounds to their identified targets are often verified through docking studies and molecular dynamics simulations based upon previously determined protein structures^18^. Thus, the greater the number of high-resolution structures available for these modeling studies, the better the odds of identifying potent inhibitors and antibiotics through rational drug design, as opposed to random hits from a large chemical screen. Concurrent with this, the development of innovative biophysical and biochemical methods to produce high-quality structural data is critical to help identify and characterize unique targets for drug development.

### Targeting efflux pumps to mitigate antibiotic resistance

**Figure 1.**
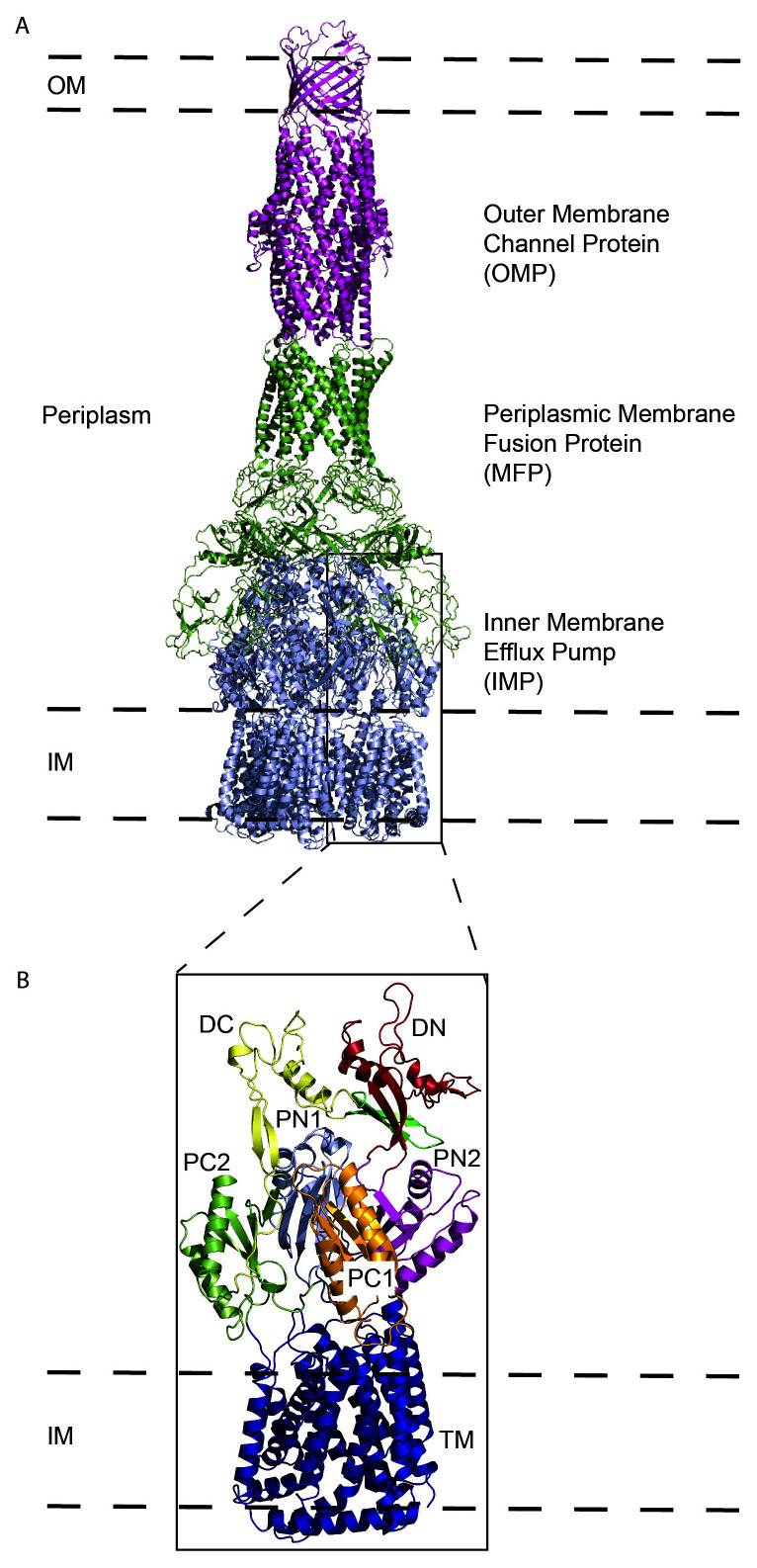
General structure of trimeric RND efflux systems. (**A**) The components of a tripartite efflux system (adapted from Protein Data Bank ID 5O66) visualized in side view with the inner membrane pump (IMP, blue), membrane fusion protein (MFP, green) and outer membrane protein (OMP, purple). Pictured is the IMP:MFP:OMP subunit ratio of 3:6:3, which is the most common assembly pattern. The outer membrane (OM) and inner membrane (IM) are designated by dashed lines. (**B**) A magnified view of one subunit of an RND-type inner membrane pump. Periplasmic subunits are designated DC (yellow), DN (red), PN1 (light blue), PN2 (purple), PC1 (orange), and PC2 (green). The transmembrane (TM) and membrane-associated helices are designated blue. RND, resistance–nodulation–cell division.

Drug-resistant infections are the result, in part, of the alarming frequency in which bacteria can alter their genomes. Mutations^19^, as well as mobile genetic elements^20,21^, are mechanisms that allow bacteria to acquire antibiotic resistance. These genetic changes often decrease the permeability of the outer membrane of the cell as well as increase the rate at which antibiotics are expelled^22^, and the latter is principally regulated through the function of bacterial efflux systems. These efflux systems are critical for survival by removing harmful agents detrimental to the cell^23^. Additionally, in several infectious bacteria such as *Mycobacterium tuberculosis*, these exporters are also designed to transport lipid from the interior of the cell to the outer membrane for membrane biogenesis^24^. Among various types of efflux systems, members of the resistance–nodulation–cell division (RND) superfamily are the most important in mediating antibiotic resistance in Gram-negative pathogens^25^. The traditional architecture of an RND-type bacterial efflux system consists of an outer membrane channel protein (OMP), a periplasmic membrane fusion protein (MFP), and an inner membrane efflux pump (IMP) (Figure 1A). The canonical structure of the RND-type IMP consists of 12 transmembrane helices that span the inner membrane of Gram-negative bacteria and several periplasmic domains that occupy about a third of the length of the periplasmic space (Figure 1B). Its main function is to capture substrates (antibiotics such as tetracyclines, macrolides, and aminoglycosides) from the outer leaflet of the inner membrane or periplasm (or both) and transport them to the OMP for export, with the proton motive force the energy provider to facilitate substrate translocation^25,26^. A subset of these RND-type inner membrane pumps is also responsible for the transport of lipids to the outer membrane to promote membrane biosynthesis and stability. Therefore, the development of compounds that inhibit these critical transporters represents a novel and potent strategy to combat MDR, XDR, and TDR bacterial infections.

## Technical and technological advances for membrane structural biology

To date, most of the structural and functional information obtained regarding the RND transporters has been from X-ray crystallographic and biochemical studies. Our knowledge of the three-dimensional (3D) molecular orientation, assembly states, and transport mechanisms of this transporter superfamily has greatly advanced since the first published structure of AcrB, the IMP from the AcrAB-TolC efflux system^27^, almost 20 years ago. Since then, the structures of several similar yet distinct RND IMPs have been elucidated (Table 1). These data have substantiated the importance of the IMPs and have provided valuable information on the binding and transport of their substrates. Solved IMP structures, in combination with large *in silico* chemical libraries, allow docking tools such as AutoDock Vina^28^, Schrödinger Glide^29,30^, and UCSF Dock^31^ to effectively identify and assess binding capabilities of potential inhibitory compounds. One limitation, however, of using X-ray crystallography for 3D protein reconstruction is that the method requires the production of a static crystal lattice, providing only a singular snapshot of these IMPs, forcing us to piece together mechanistic details of substrate transport. To help overcome this, molecular dynamics simulations such as protein in atomistic details coupled with coarse‐grained environment (PACE) have been employed. This technique was successful in modeling conformational changes of AcrB (the critical IMP from *E. coli*) upon indole transport and may be an effective tool to model the transport mechanisms of IMP inhibitors^32^.

**Table 1.** Solved structures of the inner membrane protein component of the RND-type of bacterial efflux system.

Organism	Inner membrane protein	Year	Protein Data Bank ID	Method
*Escherichia coli*	AcrB	2002 2006	1IWG^27^ 2DHH^39^	X-ray X-ray
*Pseudomonas aeruginosa*	MexB	2009	2V50^40^	X-ray
*E. coli*	CusA	2010	3K07^41^, 3KSS^41^, 3KSO^41^	X-ray
*Cupriavidus metallidurans*	ZneA	2013	4K0E^42^, 4K0J^42^	X-ray
*Neisseria gonorrhoeae*	MtrD	2014 2020	4MT1^43^ 6VKS^44^, 6VKT^44^	X-ray Cryo-EM
*Campylobacter jejuni*	CmeB	2017	5LQ3^45^, 5T0O^45^	X-ray
*Burkholderia multivorans*	HpnN	2017	5KHN^46^, 5KHS^46^	X-ray
*Acinetobacter baumannii*	AdeB	2019	6OWS^47^	Cryo-EM
*Mycobacterium smegmatis*	MmpL3	2019 2019	6OR2^48^ 6AJF^49^	X-ray X-ray

Cryo-EM, cryo-electron microscopy; RND, resistance–nodulation–cell division.

Despite these efforts, the difficulty of producing high-quality crystals suitable for structural determination of membrane proteins remains a significant challenge. To solve these structures via crystallography, investigators typically need to start with a high concentration of mostly pure protein^33^. Many times, the concentrations of isolated membrane proteins from bacterial or eukaryotic expression systems are insufficient to produce high-quality well-ordered crystals, while solubilization of the cell membrane for protein extraction often leads to impurities that significantly hinder the crystallization process^34^. Historically, a detergent-based buffer has been used to facilitate removal of membrane proteins from their cellular membrane environment. This is not a trivial step, as both the type and concentration of detergent need to be experimentally determined with the overall goal to extract from the membrane a soluble protein in its functional state. Often, this trial-and-error method is unsuccessful. Additionally, detergent-based extraction eliminates the native lipid bilayer that may be required to maintain the protein in its native conformation^34^. As an alternative, lipid bilayer mimetics such as bicelles^35^, lipid cubic phase^36^ and nanodiscs derived from native cell membrane nanoparticle systems (NCMNS)^37^, and styrene maleic-acid lipid particles (SMALPs)^38^ are being developed and successfully employed to help determine protein structures in their native lipid-associated state. These detergent-free systems may be beneficial for the continued study of RND transporters not only with X-ray crystallography but also with additional methods such as cryo-electron microscopy (cryo-EM)^50^, X-ray free-electron laser (XFEL)^51^, and other biophysical techniques^34^.

## Emergence of cryo-electron microscopy as a powerful tool to study membrane complexes

X-ray structures essentially portray a protein in a rigid conformation, bound by the energetics and constraints of the crystal lattice. These constraints can introduce structural artifacts, particularly for protein–ligand interactions and for the spatial orientation of multi-protein assemblies^52^. Owing to these and other limitations, how and where substrates bind the RND IMPs and how complete RND transporter systems assemble can be difficult to structurally observe solely on the basis of X-ray crystallography. The emergence of single-particle cryo-EM has provided us with a promising alternative and allows many of the limitations of X-ray crystallography to be surpassed. Improved electron detectors and image processors have empowered cryo-EM to develop from a technique that provides “blob-like” low-resolution structures to the ability to compete with X-ray crystallography by solving high-resolution structures of large protein complexes in their native conformation^53,54^. In single-particle cryo-EM, images of individual proteins or protein complexes are collected and processed to generate 3D reconstructions^55^. There are at least two major advantages: (1) The amount of protein required for cryo-EM is substantially less than that for X-ray crystallography. This eliminates many of the problems often encountered when trying to produce large quantities of recombinant protein. (2) Cryo-EM has the ability to visualize more than one conformational state in a sample. Since proteins and protein complexes greater than 100 kDa are well suited for this technique, this makes RND transporters ideal protein targets for this method. This is highlighted by the seminal work detailing the structural assembly of the *E. coli* AcrAB-TolC^56^ and the *Pseudomonas aeruginosa* MexAB-OprM^57^ tripartite transport systems by cryo-EM. Cryo-EM also has the potential to detect substrates bound at multiple locations during the transport process, through the capture of intermediate states. Indeed, we recently used cryo-EM to solve the structure of the *A. baumannii* IMP component of the AdeABC efflux transporter, AdeB^47^.

### The ability to solve protein–antibiotic complexes

**Figure 2.**
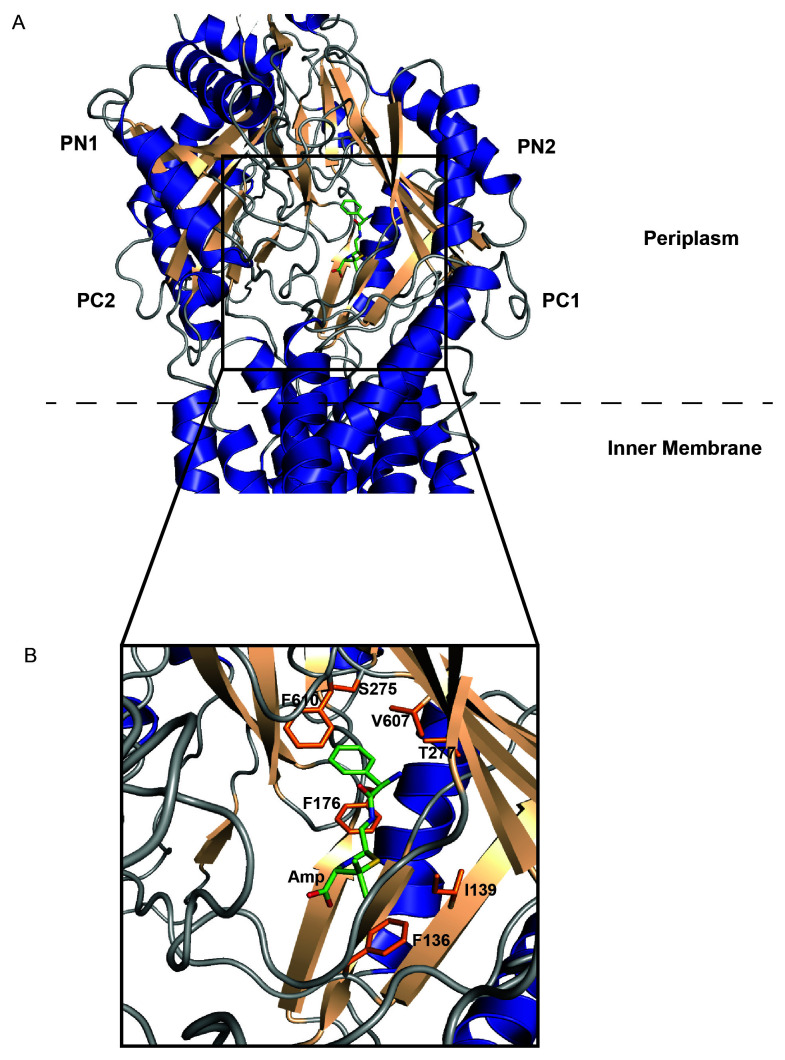
Antibiotic-bound cryo-EM structure of the *Neisseria gonorrhoeae* RND-type inner membrane pump, MtrD (adapted from Protein Data Bank ID 6VKS). (**A**) α-helices (blue), β-sheets (wheat), and loops (gray) depict the overall secondary structure of MtrD. A hydrolyzed, decarboxylated ampicillin molecule (green) is bound deep within the cavity formed by the orientation of the periplasmic domains PC1, PC2, PN1, and PN2. The inner membrane–periplasm lipid boundary is represented by a dashed line. (**B**) A magnified view of the ampicillin-binding region. Important amino acid side chains involved in substrate recognition/stabilization are shown in orange. Amp, hydrolyzed, decarboxylated ampicillin; cryo-EM, cryo-electron microscopy; RND, resistance–nodulation–cell division.

Through cryo-EM, we also determined two structures of the gonococcal MtrD transporter. We were able to elucidate how this multidrug efflux transporter specifically interacts with substrates in a more straightforward manner as we did not need to optimize crystallization conditions suitable for crystallizing MtrD bound with drugs. In *Neisseria gonorrhoeae*, the causative agent of the sexually transmitted infection gonorrhea, the multiple transferable resistance efflux system, MtrCDE, exports a wide variety of diverse antimicrobial agents from the cell, and its expression is a major contributor to β-lactam and macrolide resistance^58^. The inner membrane efflux transporter, MtrD, is responsible for the recognition and transport of substrates in concert with the periplasmic MFP MtrC and OMP MtrE. Therefore, targeting MtrD is a viable strategy to increase the potency of antibiotics in order to eliminate gonococcal infection. Cryo-EM was used to successfully determine structures of the MtrD efflux transporter, carrying a mosaic-like sequence, in the presence of bound antibiotics^44^. These structures enabled us to identify important residues for drug recognition, and several of these residues were independently verified *in vivo*^59^, as well as modes of MtrD–drug interactions. The drug molecules were found to bind deeply at the distal drug-binding site in the periplasmic domain of MtrD (Figure 2A). Important residues that stabilize antibiotic-MtrD binding were identified (Figure 2B) and a mechanism of substrate transport was able to be postulated. It is likely that antibiotics enter the channel from the periplasmic cleft created by domains PC1 and PC2, then sequentially bind the proximal and distal binding sites. These binding sites guide substrate movement through the channel and the eventual release to the OMP for export from the cell. Through this cryo-EM structural approach coupled with genetic studies, we also identified that the conserved charged amino acids R714 and E823 are critical for the recognition of macrolides and provide clinical non-susceptibility to azithromycin (azithromycin and ceftriaxone are recommended as the first choice for dual treatment of gonorrhea^60^). Interestingly, a global meta-analytical approach was used to analyze 4,852 clinical isolates. The study found that mutations at positions 714 and 823 of MtrD in these isolates led to azithromycin resistance well above the Clinical and Laboratory Standards Institute (CLSI) azithromycin non-susceptibility threshold^61^, confirming our hypothesis that amino acid changes at MtrD positions 714 and 823 could lead to clinically significant levels of azithromycin non-susceptibility resistance. Because of the high quality of the structural data, we were also able to detail the proton transfer process within the proton relay network, which provides the proton motive force to power up this multidrug efflux transporter. Taken together, these structural studies allowed the correlation of spontaneous resistance mutations within MtrD to specific locations within the protein and can aid in the design of new and more effective inhibitory compounds to obstruct the principal multidrug efflux mechanism in *N. gonorrhoeae*.

### Cryo-electron microscopy as a tool to study the membrane proteome

**Figure 3.**
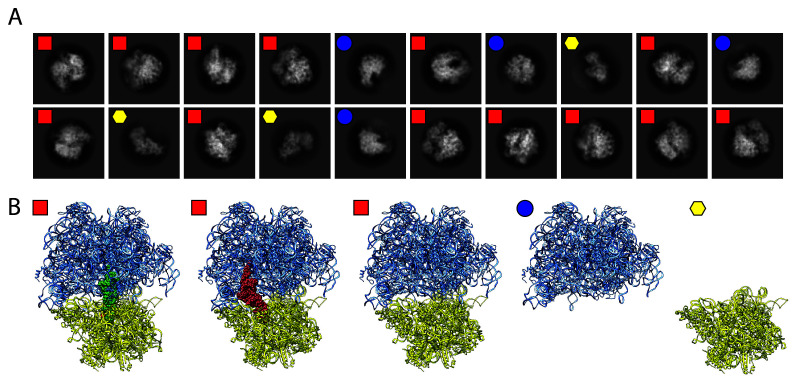
Ribosomal structures determined by single particle cryo-EM. (**A**) Two-dimensional class averages obtained from a single cryo-EM experiment. Ribosomes were isolated from the *Acinetobacter baumannii* bacterium and flash-frozen onto a cryogenic grid. Shown are three separate classes that differentiate the 70S complex (red squares) from the individual 50S (blue circles) and the 30S (yellow hexagons) subunits. (**B**) Further computational sorting and analysis revealed three separate states of the intact 70S ribosome: tRNA bound at the P-site (green spheres), the E-site (red spheres), or empty. cryo-EM, cryo-electron microscopy.

The purification of membrane proteins to near homogeneity can be a difficult and laborious process. This is especially challenging when trying to express eukaryotic membrane proteins using either current cell culture systems or non-eukaryotic expression methods. These purifications can lead to dilute, impure samples. Although low concentration and purity are not necessarily detrimental for reconstructing small (<500 kDa) water-soluble protein complexes^62^, they are often roadblocks for obtaining high-quality crystals of larger complexes, especially those that associate with the membrane. In many cases, cryo-EM is able to help overcome these homogeneity and purity problems. After data collection and initial image processing, *in silico* purification of the particle set allows separation of particle classes based of the results of 2D and 3D *ab initio* classifications. This process helps to separate different views of the target protein from those that belong to impurities, which allows map building of the protein of interest. While methods have been developed to build maps of multiple water-soluble protein complexes from a heterogeneous, partially purified sample^63–69^, we are currently developing an iterative methodology to handle impure, heterogeneous samples for structural determination of RND transporters and other membrane-bound complexes by cryo-EM. To test our strategy, we determined cryo-EM structures of the *A. baumannii* 70S ribosome^70^. The intact 70S complex as well as the individual 30S and 50S subunits were able to be identified in distinct 2D class averages from a single cryogenic sample (Figure 3A). Using 3D variability analysis, we were able to detect the inter-particle motions of the 70S ribosome in different tRNA-bound states (Figure 3B), as well as visualize the ribosome in various conformations upon the introduction of ribosomal-specific substrates, thus allowing an enhanced understanding of the structural dynamics of ribosomal function from one sample. These techniques can be effectively extended to analyze membrane isolates. Indeed, with cryo-EM, it will soon be possible to simultaneously solve structures of several bacterial membrane proteins to near atomic resolution^67^. Non-homogeneous sample preparations will no longer be restrictive for these membrane isolates, and significant data can be obtained from a single, heterogeneous protein sample.

In addition to the generation of high-quality structures of protein–substrate complexes that are challenging to solve with X-ray crystallography, cryo-EM has the potential to be used in a broader scope as a conduit to structural systems proteomics. Many cellular machineries consist of protein complexes and enzymatic pathways that span both the inner and outer membranes of Gram-negative bacteria as well as eukaryotic mitochondria. With the advancements of cryo-EM as well as the recent development of native mass spectrometry^71^, it is now possible to study these proteins within intact membranes. It is not unrealistic to foresee how multiple structures of a complex or enzymatic chain can be solved simultaneously with the help of these cutting-edge technologies. This will enable the identification of important protein–protein contacts and help discern how these proteins assemble into a functional complex. The end result is the development of an integrated systems approach to structural biology that will greatly aid in elucidating the membrane proteome. This, in turn, will significantly advance our knowledge of how cells develop multidrug resistance and shed light on new membrane complexes and transport systems as potential targets for the development of new inhibitors or biocides or both.

## Future prospective

The field of structural biology has developed into a powerful tool in the fight against drug-resistant bacterial infections. X-ray crystallography has been the standard for protein structural determination for decades and the foundation on which structure-based drug design has been built. With the advancement of additional techniques, such as cryo-EM, cryo-electron tomography (cryo-ET)^72^, microcrystal electron diffraction (micro-ED)^73^, and XFEL^51^, the ability to generate structural data has never been greater. Protein structures in their native state as well as structures of protein–ligand and protein–protein complexes are becoming easier to determine. It is also possible to thoroughly analyze the dynamics of biomacromolecular systems via these state-of-the-art technologies. This is especially beneficial for the study of bacterial efflux systems, whose inhibition holds great promise as a method to increase the potency of current antibiotic treatment regimens.

Through cryo-EM, it is possible for the IMPs in *E. coli*, *N. gonorrhoeae*, *A. baumannii*, and *Campylobacter jejuni*, among others, to be determined in their solution state, with and without bound substrate. Combined, these structures will allow a more complete description of how these pumps recognize and transport substrates. The attractiveness of these IMPs as potential drug targets is highlighted in a recent study that identified 42 new efflux pump inhibitors from a screen of about 50,000 diverse compounds^74^. The ability to determine how and where these substrates interact within the pumps would greatly enhance the optimization efforts required to produce more potent inhibitors, as these compounds have been shown to block the substrate transport pathway at multiple locations. This example highlights the ability of structural biology to be a cornerstone for cooperative scientific techniques used in conjunction for inhibitor design and drug development.

Although there has been significant success in solving individual components of the RND transporter superfamily, it has been a formidable task to obtain structural information of a completely assembled tripartite efflux complex, which spans the entire bacterial cell envelope. The most successful visualizations have been for the AcrAB-TolC efflux system^56^ by cryo-EM and its *in situ* structure using cryo-ET^75^. Through the utility of these powerful techniques combined with optimized sample preparations, it is likely that additional complete transport machineries (Figure 1) will be able to be viewed as a complete complex as opposed to each component solved separately. This improved methodology will extend to other important biological systems such as electron transport chains, respiratory complexes, and toxins^76–78^. The intimate understanding of these transporters, porins, channels, and other proteins that make up the membrane proteome will greatly enhance the ability to identify new targets and design novel compounds to combat the rapid evolution of MDR strains of bacteria.

## References

[1] Centers for Disease Control and Prevention (CDC): Antibiotic Resistance Threats in the United States, 2019. CDC. 2019. 10.15620/cdc:82532

[2] Antibiotic Resistance. World Health Organization. Geneva: WHO. 2020. Reference Source

[3] Wang CH Hsieh YH Powers ZM: Defeating Antibiotic-Resistant Bacteria: Exploring Alternative Therapies for a Post-Antibiotic Era. *Int J Mol Sci.* 2020; 21(3): 1061. 10.3390/ijms21031061 32033477 PMC7037027

[4] Sandhu P Akhter Y: Evolution of structural fitness and multifunctional aspects of mycobacterial RND family transporters. *Arch Microbiol.* 2018; 200(1): 19–31. 10.1007/s00203-017-1434-6 28951954

[5] Bunsow E Los-Arcos I Martin-Gómez MT: Donor-derived bacterial infections in lung transplant recipients in the era of multidrug resistance. *J Infect.* 2020; 80(2): 190–196. 10.1016/j.jinf.2019.12.006 31843689

[6] Peleg AY Seifert H Paterson DL: *Acinetobacter baumannii*: Emergence of a successful pathogen. *Clin Microbiol Rev.* 2008; 21(3): 538–82. 10.1128/CMR.00058-07 18625687 PMC2493088

[7] Salgado-Camargo AD Castro-Jaimes S Gutierrez-Rios RM: Structure and Evolution of *Acinetobacter baumannii* Plasmids. *Front Microbiol.* 2020; 11: 1283. 10.3389/fmicb.2020.01283 32625185 PMC7315799

[8] Antunes LCS Visca P Towner KJ: *Acinetobacter baumannii*: Evolution of a global pathogen. *Pathog Dis.* 2014; 71(3): 292–301. 10.1111/2049-632X.12125 24376225

[9] Matuszewska M Murray GGR Harrison EM: The Evolutionary Genomics of Host Specificity in *Staphylococcus aureus*. *Trends Microbiol.* 2020; 28(6): 465–77. 10.1016/j.tim.2019.12.007 31948727

[10] Hiramatsu K Cui L Kuroda M Ito T: The emergence and evolution of methicillin-resistant *Staphylococcus aureus*. *Trends Microbiol.* 2001; 9(10): 486–93. 10.1016/s0966-842x(01)02175-8 11597450

[11] Lakhundi S Zhang K: Methicillin-Resistant *Staphylococcus aureus*: Molecular Characterization, Evolution, and Epidemiology. *Clin Microbiol Rev.* 2018; 31(4): e00020–18. 10.1128/CMR.00020-18 30209034 PMC6148192

[12] Lenski RE Rose MR Simpson SC: Long-Term Experimental Evolution in *Escherichia coli*. I. Adaptation and Divergence During 2,000 Generations. *Am Nat.* 1991; 138(6): 1315–41. 10.1086/285289

[13] Sniegowski PD Gerrish PJ Lenski RE: Evolution of high mutation rates in experimental populations of *E. coli*. *Nature.* 1997; 387(6634): 703–5. 10.1038/42701 9192894

[14] Choe D Lee JH Yoo M: Adaptive laboratory evolution of a genome-reduced *Escherichia coli*. *Nat Commun.* 2019; 10(1): 935. 10.1038/s41467-019-08888-6 30804335 PMC6389913

[15] Baumgartner M Bayer F Pfrunder-Cardozo KR: Resident microbial communities inhibit growth and antibiotic-resistance evolution of *Escherichia coli* in human gut microbiome samples. *PLoS Biol.* 2020; 18(4): e3000465. 10.1371/journal.pbio.3000465 32310938 PMC7192512

[16] Salcher S Spoden G Hagenbuchner J: A drug library screen identifies Carbenoxolone as novel FOXO inhibitor that overcomes FOXO3-mediated chemoprotection in high-stage neuroblastoma. *Oncogene.* 2020; 39(5): 1080–97. 10.1038/s41388-019-1044-7 31591479 PMC6989399

[17] Schimmel K Jung M Foinquinos A: Natural Compound Library Screening Identifies New Molecules for the Treatment of Cardiac Fibrosis and Diastolic Dysfunction. *Circulation.* 2020; 141(9): 751–67. 10.1161/CIRCULATIONAHA.119.042559 31948273 PMC7050799

[18] Vargiu AV Nikaido H: Multidrug binding properties of the AcrB efflux pump characterized by molecular dynamics simulations. *Proc Natl Acad Sci U S A.* 2012; 109(50): 20637–42. 10.1073/pnas.1218348109 23175790 PMC3528587

[19] Moon DC Seol SY Gurung M: Emergence of a new mutation and its accumulation in the topoisomerase IV gene confers high levels of resistance to fluoroquinolones in *Escherichia coli* isolates. *Int J Antimicrob Agents.* 2010; 35(1): 76–9. 10.1016/j.ijantimicag.2009.08.003 19781915

[20] Carraro N Matteau D Luo P: The master activator of IncA/C conjugative plasmids stimulates genomic islands and multidrug resistance dissemination. *PLoS Genet.* 2014; 10(10): e1004714. 10.1371/journal.pgen.1004714 25340549 PMC4207636

[21] Gillings MR: Integrons: Past, present, and future. *Microbiol Mol Biol Rev.* 2014; 78(2): 257–77. 10.1128/MMBR.00056-13 24847022 PMC4054258

[22] Maslov DA Shur KV Vatlin AA: MmpS5-MmpL5 Transporters Provide *Mycobacterium smegmatis* Resistance to imidazo[1,2- *b*][1,2,4,5]tetrazines. *Pathogens.* 2020; 9(3): 166. 10.3390/pathogens9030166 32121069 PMC7157563

[23] Nikaido H: Structure and mechanism of RND-type multidrug efflux pumps. *Adv Enzymol Relat Areas Mol Biol.* 2011; 77: 1–60. 10.1002/9780470920541.ch1 21692366 PMC3122131

[24] Xu Z Meshcheryakov VA Poce G: MmpL3 is the flippase for mycolic acids in mycobacteria. *Proc Natl Acad Sci U S A.* 2017; 114(30): 7993–8. 10.1073/pnas.1700062114 28698380 PMC5544280

[25] Nikaido H: RND transporters in the living world. *Res Microbiol.* 2018; 169(7–8): 363–71. 10.1016/j.resmic.2018.03.001 29577985 PMC6151166

[26] Nikaido H Takatsuka Y: Mechanisms of RND multidrug efflux pumps. *Biochim Biophys Acta.* 2009; 1794(5): 769–81. 10.1016/j.bbapap.2008.10.004 19026770 PMC2696896

[27] Murakami S Nakashima R Yamashita E: Crystal structure of bacterial multidrug efflux transporter AcrB. *Nature.* 2002; 419(6907): 587–93. 10.1038/nature01050 12374972

[28] Trott O Olson AJ: AutoDock Vina: Improving the speed and accuracy of docking with a new scoring function, efficient optimization, and multithreading. *J Comput Chem.* 2010; 31(2): 455–61. 10.1002/jcc.21334 19499576 PMC3041641

[29] Friesner RA Banks JL Murphy RB: Glide: A new approach for rapid, accurate docking and scoring. 1. Method and assessment of docking accuracy. *J Med Chem.* 2004; 47(7): 1739–49. 10.1021/jm0306430 15027865

[30] Halgren TA Murphy RB Friesner RA: Glide: A new approach for rapid, accurate docking and scoring. 2. Enrichment factors in database screening. *J Med Chem.* 2004; 47(7): 1750–9. 10.1021/jm030644s 15027866

[31] Allen WJ Balius TE Mukherjee S: DOCK 6: Impact of new features and current docking performance. *J Comput Chem.* 2015; 36(15): 1132–56. 10.1002/jcc.23905 25914306 PMC4469538

[32] Jewel Y van Dinh Q Liu J: Substrate-dependent transport mechanism in AcrB of multidrug resistant bacteria. *Proteins.* 2020; 88(7): 853–64. 10.1002/prot.25877 31998988 PMC7292753

[33] Ishchenko A Abola EE Cherezov V: Crystallization of Membrane Proteins: An Overview. *Methods Mol Biol.* 2017; 1607: 117–41. 10.1007/978-1-4939-7000-1_5 28573571

[34] Guo Y: Be Cautious with Crystal Structures of Membrane Proteins or Complexes Prepared in Detergents. * Crystals (Basel).* 2020; 10(2): 86. 10.3390/cryst10020086 32494365 PMC7269168

[35] Faham S Bowie JU: Bicelle crystallization: A new method for crystallizing membrane proteins yields a monomeric bacteriorhodopsin structure. *J Mol Biol.* 2002; 316(1): 1–6. 10.1006/jmbi.2001.5295 11829498

[36] Caffrey M: A comprehensive review of the lipid cubic phase or *in meso* method for crystallizing membrane and soluble proteins and complexes. *Acta Crystallogr F Struct Biol Commun.* 2015; 71(Pt 1): 3–18. 10.1107/S2053230X14026843 25615961 PMC4304740

[37] Qiu W Fu Z Xu GG: Structure and activity of lipid bilayer within a membrane-protein transporter. *Proc Natl Acad Sci U S A.* 2018; 115(51): 12985–90. 10.1073/pnas.1812526115 30509977 PMC6304963

[38] Parmar M Rawson S Scarff CA: Using a SMALP platform to determine a sub-nm single particle cryo-EM membrane protein structure. *Biochim Biophys Acta Biomembr.* 2018; 1860(2): 378–83. 10.1016/j.bbamem.2017.10.005 28993151 PMC5780298

[39] Murakami S Nakashima R Yamashita E: Crystal Structures of a Multidrug Transporter Reveal a Functionally Rotating Mechanism. *Nature.* 2006; 443(7108): 173–179. 10.1038/nature05076 16915237

[40] Sennhauser G Bukowska MA Briand C: Crystal Structure of the Multidrug Exporter MexB from Pseudomonas Aeruginosa. *J Mol Biol.* 2009; 389(1): 134–145. 10.1016/j.jmb.2009.04.001 19361527

[41] Long F Su C-C Zimmermann MT: Crystal Structures of the CusA Efflux Pump Suggest Methionine-Mediated Metal Transport. *Nature.* 2010; 467(7314): 484–488. 10.1038/nature09395 20865003 PMC2946090

[42] Pak JE Ekendé EN Kifle EG: Structures of Intermediate Transport States of ZneA, a Zn(II)/Proton Antiporter. *Proc Natl Acad Sci U S A.* 2013; 110(46): 18484–18489. 10.1073/pnas.1318705110 24173033 PMC3832018

[43] Bolla JR Su C-C Do SV: Crystal Structure of the *Neisseria Gonorrhoeae* MtrD Inner Membrane Multidrug Efflux Pump. *PLoS One.* 2014; 9: e97903. 10.1371/journal.pone.0097903 24901477 PMC4046932

[44] Lyu M Moseng MA Reimche JL: Cryo-EM Structures of a Gonococcal Multidrug Efflux Pump Illuminate a Mechanism of Drug Recognition and Resistance. *mBio.* 2020; 11(1): e00996–20. 10.1128/mBio.00996-20 32457251 PMC7251214

[45] Su C-C Yin L Kumar N: Structures and Transport Dynamics of a *Campylobacter Jejuni* Multidrug Efflux Pump. *Nat Commun.* 2017; 8(1): 171. 10.1038/s41467-017-00217-z 28761097 PMC5537355

[46] Kumar N Su C-C Chou TH: Crystal Structures of the *Burkholderia Multivorans* Hopanoid Transporter HpnN. *Proc Natl Acad Sci.* 2017; 114(25): 6557–6562. 10.1073/pnas.1619660114 28584102 PMC5488925

[47] Su CC Morgan CE Kambakam S: Cryo-Electron Microscopy Structure of an *Acinetobacter baumannii* Multidrug Efflux Pump. *mBio.* 2019; 10(4): e01295–19. 10.1128/mBio.01295-19 31266873 PMC6606808

[48] Su C-C Klenotic PA Bolla JR: MmpL3 Is a Lipid Transporter That Binds Trehalose Monomycolate and Phosphatidylethanolamine. *Proc Natl Acad Sci.* 2019; 116(23): 11241–11246. 10.1073/pnas.1901346116 31113875 PMC6561238

[49] Zhang B Li J Yang X: Crystal Structures of Membrane Transporter MmpL3, an Anti-TB Drug Target. *Cell.* 2019; 176(3): 636–648. e13. 10.1016/j.cell.2019.01.003 30682372

[50] Efremov RG Gatsogiannis C Raunser S: Lipid Nanodiscs as a Tool for High-Resolution Structure Determination of Membrane Proteins by Single-Particle Cryo-EM. * Methods Enzymol.* 2017; 594: 1–30. 10.1016/bs.mie.2017.05.007 28779836

[51] Rossbach J Schneider JR Wurth W: 10 years of pioneering X-ray science at the Free-Electron Laser FLASH at DESY. *Physics Reports.* 2019; 808: 1–74. 10.1016/j.physrep.2019.02.002

[52] Kobe B Guncar G Buchholz R: Crystallography and protein-protein interactions: Biological interfaces and crystal contacts. *Biochem Soc Trans.* 2008; 36(Pt 6): 1438–41. 10.1042/BST0361438 19021571

[53] Yip KM Fischer N Paknia E: Atomic-resolution protein structure determination by cryo-EM. *Nature.* 2020; 587(7832): 157–61. 10.1038/s41586-020-2833-4 33087927

[54] Nakane T Kotecha A Sente A: Single-Particle Cryo-EM at Atomic Resolution. *BioRxiv.* 2020. 10.1101/2020.05.22.110189 PMC7611073 33087931

[55] Cheng Y Grigorieff N Penczek PA: A primer to single-particle cryo-electron microscopy. *Cell.* 2015; 161(3): 438–49. 10.1016/j.cell.2015.03.050 25910204 PMC4409659

[56] Du D Wang Z James NR: Structure of the AcrAB-TolC multidrug efflux pump. *Nature.* 2014; 509(7501): 512–5. 10.1038/nature13205 24747401 PMC4361902

[57] Sakurai K Yamasaki S Nakao K: Crystal structures of multidrug efflux pump MexB bound with high-molecular-mass compounds. *Sci Rep.* 2019; 9(1): 4359. 10.1038/s41598-019-40232-2 30867446 PMC6416280

[58] Rouquette-Loughlin CE Reimche JL Balthazar JT: Mechanistic Basis for Decreased Antimicrobial Susceptibility in a Clinical Isolate of *Neisseria gonorrhoeae* Possessing a Mosaic-Like *mtr* Efflux Pump Locus. *mBio.* 2018; 9(6): e02281–18. 10.1128/mBio.02281-18 30482834 PMC6282211

[59] Chitsaz M Booth L Blyth MT: Multidrug Resistance in *Neisseria gonorrhoeae*: Identification of Functionally Important Residues in the MtrD Efflux Protein. *mBio.* 2019; 10(6): e02277–19. 10.1128/mBio.02277-19 31744915 PMC6867893

[60] Ison CA Hussey J Sankar KN: Gonorrhoea treatment failures to cefixime and azithromycin in England, 2010. *Euro Surveill.* 2011; 16(14): 19833. 21492528

[61] Ma KC Mortimer TD Hicks AL: Increased Antibiotic Susceptibility in Neisseria Gonorrhoeae through Adaptation to the Cervical Environment. *BioRxiv.* 2020. 10.1038/s41467-020-17980-1 PMC7431566 32807804

[62] Totir M Echols N Nanao M: Macro-to-micro structural proteomics: Native source proteins for high-throughput crystallization. *PLoS One.* 2012; 7(2): e32498. 10.1371/journal.pone.0032498 22393408 PMC3290569

[63] Han BG Dong M Liu H: Survey of large protein complexes in *D. vulgaris* reveals great structural diversity. *Proc Natl Acad Sci U S A.* 2009; 106(39): 16580–5. 10.1073/pnas.0813068106 19805340 PMC2742403

[64] Maco B Ross IL Landsberg MJ: Proteomic and electron microscopy survey of large assemblies in macrophage cytoplasm. *Mol Cell Proteomics.* 2011; 10(6): M111.008763. 10.1074/mcp.M111.008763 21406389 PMC3108843

[65] Kastritis PL O'Reilly FJ Bock T: Capturing protein communities by structural proteomics in a thermophilic eukaryote. *Mol Syst Biol.* 2017; 13(7): 936. 10.15252/msb.20167412 28743795 PMC5527848

[66] Verbeke EJ Mallam AL Drew K: Classification of Single Particles from Human Cell Extract Reveals Distinct Structures. *Cell Rep.* 2018; 24(1): 259-268.e3. 10.1016/j.celrep.2018.06.022 29972786 PMC6109231

[67] Ho CM Li X Lai M: Bottom-up structural proteomics: CryoEM of protein complexes enriched from the cellular milieu. *Nat Methods.* 2020; 17(1): 79–85. 10.1038/s41592-019-0637-y 31768063 PMC7494424

[68] Yi X Verbeke EJ Chang Y: Electron microscopy snapshots of single particles from single cells. *J Biol Chem.* 2019; 294(5): 1602–8. 10.1074/jbc.RA118.006686 30541924 PMC6364765

[69] Kirykowicz AM Woodward JD: Shotgun EM of mycobacterial protein complexes during stationary phase stress. *Curr Res Struct Biol.* 2020; 2: 204–12. 10.1016/j.crstbi.2020.09.002 PMC8244302 34235480

[70] Morgan CE Huang W Rudin SD: Cryo-electron Microscopy Structure of the *Acinetobacter baumannii* 70S Ribosome and Implications for New Antibiotic Development. *mBio.* 2020; 11(1): e03117-19. 10.1128/mBio.03117-19 31964740 PMC6974574

[71] Chorev DS Baker LA Di Wu: Protein assemblies ejected directly from native membranes yield complexes for mass spectrometry. *Science.* 2018; 362(6416): 829–834. 10.1126/science.aau0976 30442809 PMC6522346

[72] Schur FK: Toward high-resolution *in situ* structural biology with cryo-electron tomography and subtomogram averaging. *Curr Opin Struct Biol.* 2019; 58: 1–9. 10.1016/j.sbi.2019.03.018 31005754

[73] Nannenga BL Gonen T: The cryo-EM method microcrystal electron diffraction (MicroED). *Nat Methods.* 2019; 16(5): 369–379. 10.1038/s41592-019-0395-x 31040436 PMC6568260

[74] Marshall RL Lloyd GS Lawler AJ: New Multidrug Efflux Inhibitors for Gram-Negative Bacteria. *mBio.* 2020; 11(4): e01340-20. 10.1128/mBio.01340-20 32665275 PMC7360932

[75] Shi X Chen M Yu Z: In situ structure and assembly of the multidrug efflux pump AcrAB-TolC. *Nat Commun.* 2019; 10(1): 2635. 10.1038/s41467-019-10512-6 31201302 PMC6570770

[76] Tucker K Park E: Cryo-EM structure of the mitochondrial protein-import channel TOM complex at near-atomic resolution. *Nat Struct Mol Biol.* 2019; 26(12): 1158–1166. 10.1038/s41594-019-0339-2 31740857 PMC8439582

[77] Parey K Haapanen O Sharma V: High-resolution cryo-EM structures of respiratory complex I: Mechanism, assembly, and disease. *Sci Adv.* 2019; 5(12): eaax9484. 10.1126/sciadv.aax9484 31844670 PMC6905873

[78] Yamada T Yoshida T Kawamoto A: Cryo-EM structures reveal translocational unfolding in the clostridial binary iota toxin complex. *Nat Struct Mol Biol.* 2020; 27(3): 288–296. 10.1038/s41594-020-0388-6 32123390

